# Real-Time Detection of Staphylococcus Aureus Using Whispering Gallery Mode Optical Microdisks

**DOI:** 10.3390/bios6020020

**Published:** 2016-05-03

**Authors:** Hala Ghali, Hicham Chibli, Jay L. Nadeau, Pablo Bianucci, Yves-Alain Peter

**Affiliations:** 1 Department of Engineering Physics, Polytechnique Montreal, Montreal, QC, H3T 1J4, Canada; yves-alain.peter@polymtl.ca; 2 Department of Biomedical Engineering, McGill University, Montreal, QC, H3A 2B4, Canada; hicham.chibli@mcgill.ca (H.C.); jay.nadeau@mcgill.ca (J.L.N.); 3 Physics Department, Concordia University, Montreal, QC, H4B 1R6, Canada; pablo.bianucci@concordia.ca

**Keywords:** optical microdisk, surface functionalization, bacterial sensing, specific binding

## Abstract

Whispering Gallery Mode (WGM) microresonators have recently been studied as a means to achieve real-time label-free detection of biological targets such as virus particles, specific DNA sequences, or proteins. Due to their high quality (Q) factors, WGM resonators can be highly sensitive. A biosensor also needs to be selective, requiring proper functionalization of its surface with the appropriate ligand that will attach the biomolecule of interest. In this paper, WGM microdisks are used as biosensors for detection of *Staphylococcus aureus*. The microdisks are functionalized with LysK, a phage protein specific for staphylococci at the genus level. A binding event on the surface shifts the resonance peak of the microdisk resonator towards longer wavelengths. This reactive shift can be used to estimate the surface density of bacteria that bind to the surface of the resonator. The limit of detection of a microdisk with a Q-factor around 10^4^ is on the order of 5 pg/mL, corresponding to 20 cells. No binding of *Escherichia coli* to the resonators is seen, supporting the specificity of the functionalization scheme.

## 1. Introduction

Viruses and bacteria are a major cause of human disease [1]. *Staphylococcus aureus* causes serious skin and respiratory infections and food poisoning. Overuse of antibiotics has given rise to antibiotic-resistant strains such as methicillin-resistant *Staphylococcus aureus* (MRSA), which is responsible for over 60% of staphylococcal infections in hospitals [2,3]. It is crucial to find new approaches to fast diagnosis and early detection of staphylococcal infections. Real-time PCR is the fastest (about 2 h) [4] and most widespread (FDA) (United States Food and Drug Administration)-approved test for MRSA. However, this technique can be very expensive and is impractical for use on-site or in developing countries.

Other biosensing techniques such as fluorescent immunoassays require labeling of the target molecule, adding an extra step to the functionalization process. Although these methods can be very specific, especially in complex environments such as blood, they require a prior knowledge of the target molecule [5].

Therefore, it is useful to develop label-free sensors to detect the bacterium itself, rather than a fluorescent label. Whispering gallery mode microresonators have been recently studied for this purpose, considering their high quality factors that yield high sensitivity, as well as their selectivity, fast response, low cost and reproductibility [6,7,8,9]. In order to obtain good selectivity, the biosensor needs to be properly functionalized with a ligand that is specific to the biomolecule to be detected. This ligand may be an antibody, aptamer, complementary sequence, or other molecule that binds specifically to the target of interest while ideally protecting the resonator from non-specific binding.

Non-specific bacterial detection has been demonstrated in previous studies [10,11]. However, non-specific biosensors are not suited for clinical uses in complex environments such as blood or serum. In this study, we demonstrate real-time specific detection of *Staphylococcus aureus* using whispering gallery mode microdisks. Specific functionalization was achieved using LysK phage protein. LysK is an endolysin from the staphylococcal phage K that binds strongly to staphylococci. It contains three domains: an *N*-terminal cysteine, a histidine-dependent amidohydrolase/peptidase (CHAP) domain, a midprotein amidase-2 domain, and a *C*-terminal SH3b cell wall-binding domain [12]. It has previously been studied as a potential antimicrobial [13] and a biosensor [14]. The LysK-functionalized microdisks showed a concentration-dependent wavelength shift in the presence of *S. aureus*. No shift was seen with a control strain (*E. coli*), indicating specificity at least at the genus level.

## 2. Materials and Methods

### 2.1. Microdisk Fabrication

Optical microdisks were fabricated using silicon micromachining techniques [15]. The substrates used were silicon topped with an 800 nm thermal silicon dioxide layer. The first step performed was UV photolithography to generate photoresist patterns of 200 μm-diameter microdisks with AZ5214 photoresist. Patterns were then transferred to silica using buffered oxide etch (B.O.E.) of silicon dioxide. The last step was isotropic dry etching of silicon with SF${}_{6}$ gas to obtain microdisks on pedestals. Microdisks were characterized by scanning electron microscopy (Hitachi S-4700, Tokyo, Japan).

### 2.2. Plasmids and LysK Purification

Phage proteins expressed in the pET21 plasmid vector were kindly provided by David M. Donovan. The plasmid constructs [16,17] and LysK purification procedure were previously described [14]. In summary, 0.5 mg of plasmid DNA was transformed into *E. coli* BL21 (DE3) and grown overnight in modified lysogeny broth (LB) (15 g tryptone, 8 g yeast extract, 5 g NaCl per liter, pH 7.8). Colonies were picked and grown in modified LB until they reached an optical density at 600 nm (OD${}_{600}$) of 0.4–0.5. Then, protein expression was induced with 1 mM IPTG (Isopropyl *β*-D-1-thiogalactopyranoside) at 10 ${}^{\circ }$C for 20 h. The bacteria were pelleted by centrifugation (5000 rpm, 4 ${}^{\circ }$C) and the pellet was resuspended in lysis buffer (50 mM NaH${}_{2}$PO${}_{4}$, 300 mM NaCl, 10 mM imidazole, pH 8.0 and 30% glycerol) and sonicated to lyse the cells. His-tagged proteins were purified through nickel chromatography using Ni-NTA (NitriloTriacetic Acid) Superflow (Qiagen, Hilden, Germany). Supernatant was incubated with Ni-NTA matrix at 4 ${}^{\circ }$C for 1 h. Then, the column was washed once with lysis buffer and twice with wash buffer (50 mM NaH${}_{2}$PO${}_{4}$, 300 mM NaCl, 20 mM imidazole, pH 8.0 + 30% glycerol). Protein was eluted with elution buffer (50 mM NaH${}_{2}$PO${}_{4}$, 300 mM NaCl, 250 mM imidazole, pH 8.0 + 30% glycerol). The purified protein was analyzed at 10% SDS-PAGE (Sodium Dodecyl Sulfate-PolyAcrylamide Gel Electrophoresis) and visualized with Coomassie blue staining. Lytic activity of the proteins against bacterial strains was tested with a zymogram assay as follows: the mid-log growth phase cell pellet from 75 mL of bacterial culture was mixed with SDS-PAGE resolving gel. The gel was run as usual (1 h), then rinsed with dH${}_{2}$O and incubated in Tris-Buffered Saline (TBS) (10 mM Tris, 150 mM NaCl, pH 7.5) for up to 24 h until clearing in the turbid gel appeared, indicating lytic activity.

### 2.3. Surface Functionalization

The samples were cleaned using oxygen plasma or piranha etch to remove organic residues. The wafers were then immersed in an ethanol:water (95:5) solution containing 2.5% triethoxysilane-Polyethylene Glycol (PEG)-Amine (NH${}_{2}$) (Nanocs, MW = 3400) for 2 h. Samples were then thoroughly washed with ethanol and deionized water and then annealed at 110 ${}^{\circ }$C for 2 h. The PEGylated wafers were then immersed in a 2 μM LysK:2000 μM EDC (1-Ethyl-3-(3-dimethylaminopropyl)carbodiimide) (1:1000) solution in buffer (10 mM Tris.HCl pH 7.5, 150 mM NaCl, 1% glycerol) at 4 ${}^{\circ }$C overnight, and then thoroughly washed with PBS and water.

### 2.4. Optical Characterization Setup

The light from an external cavity tunable diode laser (New focus TLB-6300-LN, Newport, VA, USA) emitting at 630 nm was evanescently coupled inside the microdisk through a tapered optical fiber. Tapered fibers were fabricated by gently stretching a single mode fiber while it was heated over a flame until the tapered region reached a 2 μm diameter. A red laser was used to limit the absorption losses in aqueous media. A function generator (Agilent 33220A) fed a trigger signal to the piezo input of the laser. The signal was set to pulse with a 10 ms width, 3.5 V amplitude and 1.75 V offset. A photodetector (Newport 818-SL/CM, Newport, VA, USA) conveyed the output signal to an oscilloscope (Agilent DSO6302A) where the transmission spectrum of the resonator was observed as the laser wavelength was scanned.

### 2.5. Bacterial Strains and Maintenance

*S. aureus* strain ATCC (American Type Culture Collection) 29213 and *E. coli* strain 10798 were purchased from ATCC. *S. aureus* and *E. coli* were maintained by serial passages in LB media. For binding studies, cells were pelleted and resuspended in Tris-buffered saline (TBS) (10 mM Tris-HCl, 150 mM NaCl, pH 7.5).

## 3. Results and Discussion

### 3.1. Fabrication and Characterization

**Figure 1:**
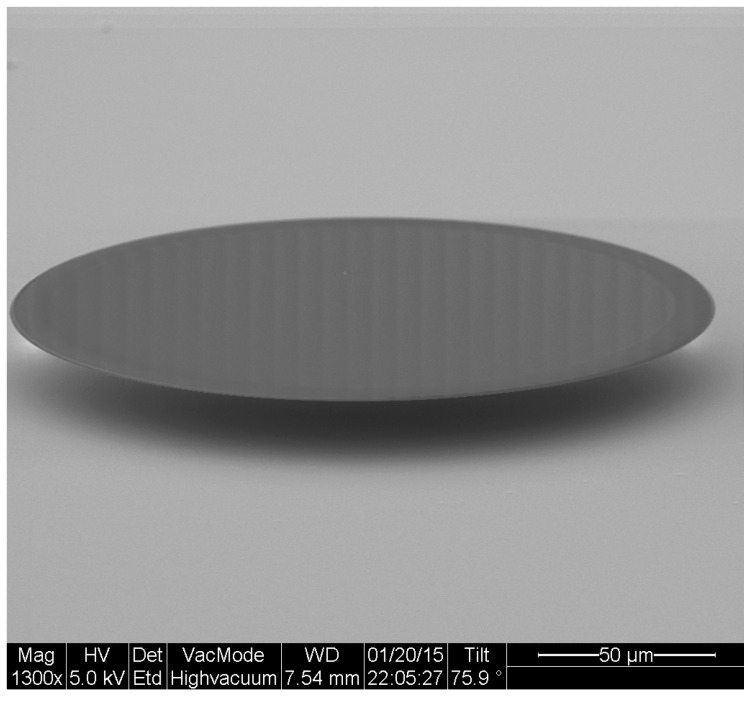
Scanning electron micrograph of an optical microdisk.

**Figure 2:**
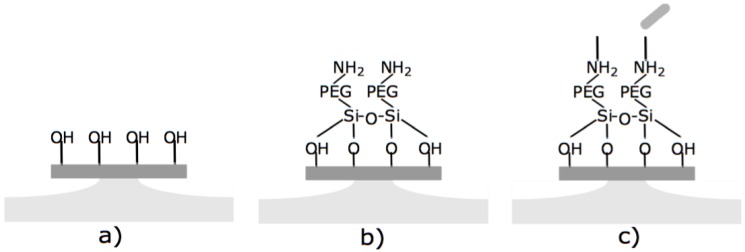
Functionalization of silica microdisks. (**a**) the silicon dioxide surface is hydroxylated with oxygen plasma; (**b**) the disk is immersed in triethoxysilane-PEG-NH${}_{2}$; (**c**) the free amines of the PEG-silane are covalently coupled to LysK using carbodiimide coupling.

An SEM image of a typical microfabricated WGM optical microdisk is shown in Figure 1. Functionalization was confirmed by ellipsometry and X-Ray Photoelectron Spectroscopy (XPS). Measurements were taken on the same silica wafers used to fabricate the microdisks and the results are detailed in [14]. The functionalized microdisks were coated with polyethylene glycol (PEG) to block any non-specific binding of bacteria to the surface of the resonator. Microdisks with 2 μM of LysK incubated in a solution of 10${}^{7}$ CFU/mL are expected to have around 60 ± 5 recognition elements per 3.3 × 10${}^{3}$ μm${}^{2}$ region [14]. A schematic of the functionalization scheme is shown in Figure 2.

### 3.2. Bacterial Binding

**Figure 3:**
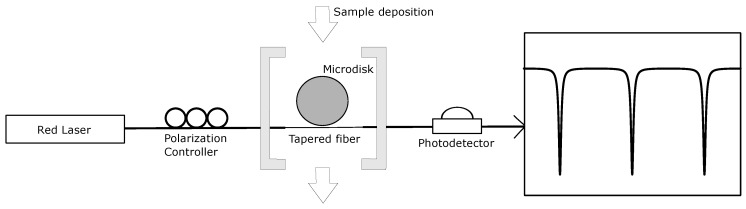
Optical characterization setup. The light from a tunable laser at 630 nm propagates through a polarization controller and is coupled inside the microdisk using a tapered fiber. The signal conveyed from the photodetector to the oscilloscope yields the transmission spectrum. Resonant modes are observed as a series of dips.

Binding experiments were performed on a functionalized 200 μm-diameter microdisk coupled to a tapered optical fiber, and the wavelength was scanned from 635.2 to 637.5 nm as shown in Figure 3. When the signal was stable, 2 μL of TBS was added to the surface of the disk using a micropipette and the transmission spectrum was observed on an oscilloscope. Addition of TBS did not lead to any wavelength shift. Keeping the same coupling position, another 2 μL of TBS containing *S. aureus* at OD${}_{600}$ = 0.4 (approximately 5 × 10${}^{9}$ CFU/mL) was added. The wavelength shift of a selected mode was observed over 30 min.

**Figure 4:**
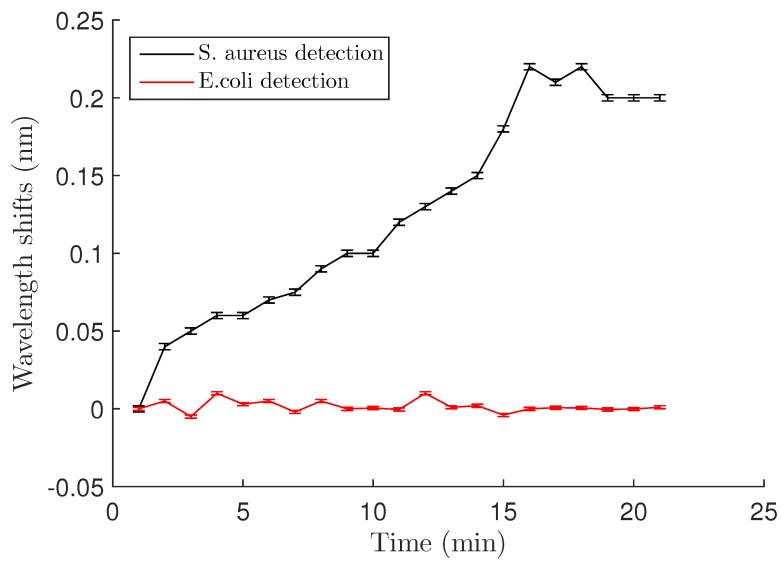
Wavelength shift *vs.* time after *S. aureus* and *E. coli* binding at a cell concentration of OD${}_{600}$ = 0.4 (5 × 10${}^{9}$ CFU/mL). After 15 min, a maximum shift is obtained and is about 0.22 nm for the Staph., whereas no shift is observed for *E. coli* detection. Error bars are standard deviations as discussed in Section 3.2 of the text.

The wavelength began to shift almost immediately after adding the bacteria, indicating rapid binding of the bacteria to the surface. The maximum wavelength shift of 0.22 ± 0.002 nm was seen after approximately 15 min (Figure 4). Uncertainties in the values resulted from laser drift (±1 pm) and the error in calculating the wavelength shift from the transmission spectra (±1 pm) . After reaching its maximum, the wavelength shift began to decrease. This is most likely because the LysK protein has strong lytic activity [12,14], so that at 30 min, a majority of the bound cells were lysed.

### 3.3. Specificity of LysK

Specificity of the PEG-LysK functionalization at the genus level was demonstrated using in a previous study fluorescent labeling [14]. Here, we confirmed this by using *E. coli* as a control with the microdisk system. *E. coli* at OD${}_{600}$ = 0.4 was applied to the resonator, and as can be seen in Figure 4, the transmission spectrum at time 0 and after 20 minutes did not change. No shifts were observed, suggesting that *E. coli* did not bind to the surface of the resonator.

When both *S. aureus* (OD${}_{600}$ = 0.4) and *E. coli* (OD${}_{600}$ = 0.4) were added to the surface of the resonator, a shift of 0.22 nm was observed, similar to the one obtained with the *S. aureus* alone. Thus, no *E. coli* attached to the resonator and only the presence of *S. aureus* contributed to the reactive shift.

### 3.4. Concentration-Dependent Shift

**Figure 5:**
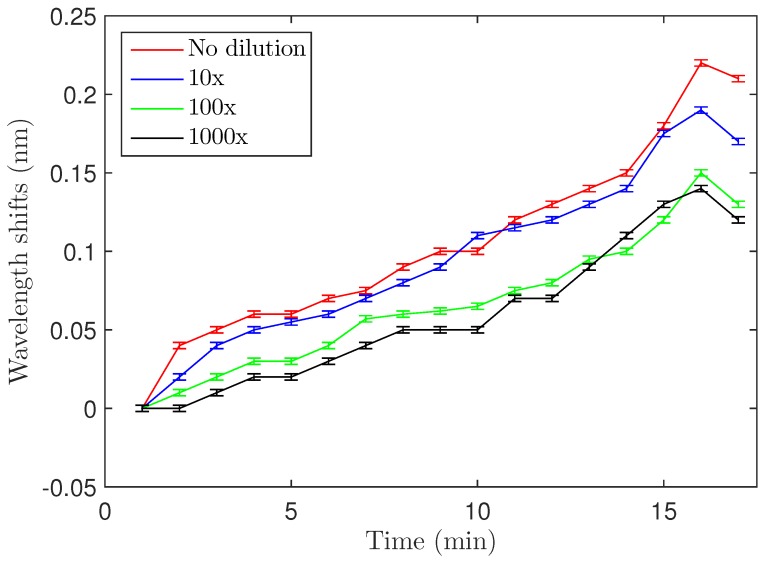
Wavelength shifts after *S. aureus* binding for four different concentrations. Error bars are standard deviations as discussed in Section 3.2 of the text.

Concentration-dependence of the shift was examined by varying the concentration of the *S. aureus* solution used for biodetection. The OD${}_{600}$ = 0.4 solution was diluted 10, 100 and 1000 fold, and Figure 5 shows the maximum wavelength shifts for the four different concentrations (also summarized in Table 1). The 100- and 1000-fold dilutions showed almost the same wavelength shift, meaning that approximately the same number of bacteria attached to the resonator in both cases. Experiments with lower concentrations could not be carried out because of the limitations due to the relatively small quality factors of the microdisks (Q ≃ 10${}^{4}$). Quality factors of the microdisks were measured after each functionalization step (addition of Si-PEG and LysK) and after biodetection. It is worth noting that the quality factor did not significantly change during the process and a value around 10,000 was found each time.

### 3.5. Bacterial Surface Density

In the previous sections, an experimental wavelength shift was found for several bacterial concentrations. A theoretical expression that links the wavelength shift to the number of bacteria that bind to the surface of the microdisk was developed in [18] and shown in Equation (1). Using this expression, it was possible to obtain an approximate number of bacteria that bound to the surface of the microdisks and contributed to the reactive shift. For example, for the solution with OD = 0.4, the shift was 0.22 nm. With a resonance wavelength of 635 nm, the surface density can be estimated to be around 8.21 × 10${}^{9}$ m${}^{-2}$:

(1) $$\frac{\delta \lambda }{\lambda }≃\frac{\alpha_{ex}\sigma_{s}}{2\epsilon_{0}\epsilon_{rs}}\left\{\frac{\left(4k_{0}\left(\sqrt{n_{d}^{2}-n_{m}^{2}}\right)\right)\;\cos^{2}\left(k_{0}\left(\sqrt{n_{d}^{2}-n_{m}^{2}}\right)\frac{h}{2}\right)}{\left[2k_{0}\left(\sqrt{n_{d}^{2}-n_{m}^{2}}\right)h+\sin\left(2k_{0}\left(\sqrt{n_{d}^{2}-n_{m}^{2}}\right)h\right)\right]}+\frac{2[J_{l}^{2}\left(k_{0}n_{m}R\right)]}{R\;[J_{l+1}^{2}\left(k_{0}n_{m}R\right)]}\right\}$$

with $\alpha_{ex}$ being the excess polarizability of the molecules binding to the resonator’s surface, $\sigma_{s}$ the surface area covered by the molecules, $\epsilon_{0}$ and $\epsilon_{rs}$ the vacuum and resonator’s relative permittivity, respectively, $k_{0}$ the wave number, $n_{d}$ and $n_{m}$ the microdisk and the solvent refractive index, respectively, *h* the thickness of the microdisk, *R* its radius and $J_{l}\left(k_{0}n_{m}R\right)$ the first kind Bessel function.

The parameters used to determine the spectral shift due to *S. aureus* binding to a 200 μm diameter silica microdisk submerged in TBS are defined in Table 2.

The surface density can be written as:

(2) $$\sigma_{s}=\frac{N}{\pi (R_{max}^{2}-r_{e}^{2})+2\pi Rh}$$

where *N* is the number of bacteria that attach to the surface, $R_{max}$ the radius of the ring where the electric field is maximum, $r_{e}$ the radius of the ring where the electric field decays to 1/e of its maximum value, *R* the radius of the microdisk and *h* its height. This suggested that 31 bacteria bound to the surface and contribute to the wavelength shift for the undiluted culture. The estimated surface coverage and the number of bacteria that bound to the surface of the microdisk for the four different concentrations used are given in Table 3.

### 3.6. Variability in Binding

Measurements were done on eight different microdisks for each concentration, and results were mean values of the experiments done for each concentration. All microdisks used had the same size of 200 μm diameter and a quality factor of around 10${}^{4}$ for the sake of comparison. In Table 4, the standard deviation (SD) of the wavelength shifts is given for the different concentrations. It can be seen that the SD increased with lower bacterial concentrations. Assuming that the microdisks used were functionalized identically, a saturation of all of the available binding sites by bacterial cells should lead to the same maximum value of the wavelength shift. This is what was seen for the highest bacterial concentration, where the wavelength shift was highly reproducible from experiment to experiment. For more dilute solutions, the bacteria will be depleted before all of the binding sites are saturated. Because some of the active protein sites will not be located on the sensitive part of the microdisk, there will be variability in the number of bound cells that contribute to the wavelength shift. Considering the four concentrations tested, the limit of detection of the biosensor for a Q-factor around 10${}^{4}$ was 5 × 10${}^{6}$ CFU/mL or 5 pg/mL.

## 4. Conclusions

In this work, we achieved real-time specific detection of *S. aureus* using whispering gallery mode microdisks functionalized with the staphylococcal-specific LysK endolysin. We showed that the resonance peak shifted towards longer wavelengths almost immediately after *S. aureus* addition, and the shift continued to increase for 15 minutes as the number of bacteria that bound to the surface of the resonator increased. Different concentrations of bacteria gave rise to different wavelength shifts, showing the dependence of the reactive shift on surface density. The limit of detection of a microdisk with a Q-factor of 10${}^{4}$ was found to be 5 pg/mL. The selectivity of the functionalization process at the genus level was also demonstrated using *E. coli*, which did not lead to any significant change of the resonance. Specificity is a very important feature of a biosensor that makes it suitable for clinical and medical use in complex environments, and we have demonstrated an important step towards it. Further studies using very low concentrations of bacteria need to be performed in order to achieve single-bacterium detection and exploit the high sensitivity of WGM resonators. The lytic effect of the LysK may be exploited by downstream sensors which could identify the lysed cells at the species or strain level, and thereby differentiate antibiotic-resistant and -sensitive strains of *S. aureus* [19].

## Figures and Tables

**Table 1:** Resonance shifts in terms of bacterial concentration.

No Dilution	Diluted 10×	Diluted 100×	Diluted 1000×
0.22 ± 0.002 nm	0.19 ± 0.002 nm	0.15 ± 0.002 nm	0.14 ± 0.002 nm

**Table 2:** Parameters of the wavelength shift equation for a microdisk.

Parameter	Definition	Value	Unit
$\alpha_{ex}$	Excess polarizability	4$\pi \epsilon_{0}$ × 2.12 × 10${}^{-20}$	m${}^{3}$
$\sigma_{s}$	Surface coverage	$\frac{N}{1.257\;\times \;10^{5}}$ (μ)	m${}^{-2}$
$\epsilon_{0}$	Vacuum permittivity	8.854 × 10${}^{-12}$	F/m
$\epsilon_{rs}$	Relative permittivity of silica	3.9	
k${}_{0}$	Wavenumber ($\frac{2\pi }{\lambda }$)	9.895 × 10${}^{-3}$	nm${}^{-1}$
n${}_{d}$	Refractive index of the microdisk	1.457	-
n${}_{m}$	Refractive index of the buffer	1.332	-
h	Thickness of the microdisk	800	nm
R	Radius of the microdisk	100 μ	m
m	Mass of one bacterium	10${}^{-12}$	g
l	Mode number ($\frac{2\pi Rn_{eff}}{\lambda }$)	1442	-

**Table 3:** Surface coverage and number of bacteria binding to the resonator for four different concentrations.

Bacterial Solution Concentration (CFU/mL)	Wavelength Shift (nm)	Surface Coverage ($m^{-2}$)	Bacteria Bound to the Surface
5 × 10${}^{9}$	0.22	8.21 ×$10^{9}$	31
5 × 10${}^{8}$	0.19	7.15 ×$10^{9}$	27
5 × 10${}^{7}$	0.15	5.56 ×$10^{9}$	21
5 × 10${}^{6}$	0.14	5.3 ×$10^{9}$	20

**Table 4:** Standard deviation of the wavelength shift for four different bacterial concentrations.

Bacterial Solution Concentration (CFU/mL)	Wavelength Shift (nm)	Standard Deviation (nm)
5 ×$10^{9}$	0.22	0.01
5 ×$10^{8}$	0.19	0.02
5 ×$10^{7}$	0.15	0.04
5 ×$10^{6}$	0.14	0.05
